# Supplementary material for: Epidemiology and disease burden of sickle cell disease in France: A descriptive study based on a French nationwide claim database
Source: PLoS One. 2021 Jul 9;16(7):e0253986. doi: 10.1371/journal.pone.0253986 (PMC8270152; doi:10.1371/journal.pone.0253986)
Supplement: S1 File — (DOCX) [file pone.0253986.s001.docx]

S1 File. EGB Description

It includes children covered under their parents’ insurance, with their reimbursement individualized from their parents’. It excludes most university students and immigrants not covered by NHI, who have specific health insurance. It should be noted that the coverage was extended from 85% to 95% between 2004 and 2016 as NHI subsets were slowly incorporated. In particular, no university students, self-employed workers or farmers were initially included. The EGB includes anonymous sociodemographic and medical characteristics and records of healthcare reimbursements since 2004. Healthcare reimbursements cover both inpatient stays and outpatient care. They only include reimbursement data with no information from medical records or results from laboratory tests or medical procedures. However, as inpatient stays are reimbursed based on Diagnosis-Related Group (DRG) claims, the diagnosis (based on ICD-10) relevant to the inpatient stay is available. Some chronic conditions as SCD are also eligible in France for special status (*Affection Longue Durée* [Long Term Illness] – ALD), which enables beneficiaries’ medical expenses to be fully covered by NHI (instead of 65% on average). ALD status information, including the associated diagnosis (based on ICD-10), is available in the EGB.
